# Evaluating reliability in wearable devices for sleep staging

**DOI:** 10.1038/s41746-024-01016-9

**Published:** 2024-03-18

**Authors:** Vera Birrer, Mohamed Elgendi, Olivier Lambercy, Carlo Menon

**Affiliations:** 1 Biomedical and Mobile Health Technology Laboratory, Department of Health Sciences and Technology, ETH Zurich, Zurich, Switzerland; 2 Department of Information Technology and Electrical Engineering, ETH Zurich, Zurich, Switzerland; 3 Rehabilitation Engineering Laboratory, Department of Health Sciences and Technology, ETH Zurich, Zurich, Switzerland

**Keywords:** Biomarkers, Public health, Biomarkers, Computer science, Biomedical engineering

## Abstract

Sleep is crucial for physical and mental health, but traditional sleep quality assessment methods have limitations. This scoping review analyzes 35 articles from the past decade, evaluating 62 wearable setups with varying sensors, algorithms, and features. Our analysis indicates a trend towards combining accelerometer and photoplethysmography (PPG) data for out-of-lab sleep staging. Devices using only accelerometer data are effective for sleep/wake detection but fall short in identifying multiple sleep stages, unlike those incorporating PPG signals. To enhance the reliability of sleep staging wearables, we propose five recommendations: (1) Algorithm validation with equity, diversity, and inclusion considerations, (2) Comparative performance analysis of commercial algorithms across multiple sleep stages, (3) Exploration of feature impacts on algorithm accuracy, (4) Consistent reporting of performance metrics for objective reliability assessment, and (5) Encouragement of open-source classifier and data availability. Implementing these recommendations can improve the accuracy and reliability of sleep staging algorithms in wearables, solidifying their value in research and clinical settings.

## Introduction

Sleep, encompassing approximately one-third of our lifespan, is a fundamental aspect of our daily activities and plays a crucial role in maintaining our health, work performance, and overall well-being^1^. Extensive research has consistently demonstrated the detrimental impact of poor sleep quality on various health conditions, including cardiovascular diseases^2^, diabetes^3^, hypertension^4^, depression^5^, immune-related diseases^6^, and cancer mortality risk^7^. As an increasing number of individuals recognize the significance of sleep quality in leading a healthy lifestyle, both sleep-related research and industries have witnessed substantial growth^8,9^.

Polysomnography (PSG) currently serves as the gold standard for sleep assessment, involving a comprehensive measurement of various physiological changes during sleep^10^. This method requires the placement of multiple sensors to monitor brain activity, heart activity, eye movements, muscle activity, blood oxygen levels, breathing patterns, body movements, snoring, and other noises. However, the complex setup and high cost associated with PSG discourage regular testing, thereby limiting its utility for accurate sleep monitoring. Patients undergoing PSG must endure the placement of numerous sensors on their bodies, intricate wiring systems, and bulky electronic devices for data transmission and storage. Additionally, PSG recordings primarily take place within specialized sleep laboratories, which are often inhospitable to natural sleep patterns^10^. Consequently, many patients experience difficulties falling asleep and do not exhibit natural sleep behavior due to the elaborate setup.

While many wearable-based algorithms focus on distinguishing between sleep and wakefulness, a comprehensive evaluation of sleep architecture and specific sleep stages is essential for proper diagnosis and treatment of sleep disorders^11^. Sleep staging provides valuable insights into the quality, characteristics, and transitions of sleep stages, enabling a more thorough understanding of sleep patterns and facilitating tailored interventions^12^.

Recent articles have summarized the use of commercially available devices for sleep monitoring, yet there is a notable gap in addressing the development of algorithms for sleep staging and the associated challenges. In response to this gap, this review aims to provide a comprehensive overview of recent advancements in wearable sensors and portable electronics, particularly focusing on innovations that enhance the comfort and usability of sleep monitoring devices by eliminating the need for adhesive, conductive gels, or cable connections. We also offer essential recommendations to guide future developments in algorithm design for wearables, targeting the accurate and reliable assessment of sleep parameters. This work is essential in improving the diagnosis and management of sleep disorders, ultimately contributing to better overall sleep health and well-being^13–15^.

## Results

### Publications

**Fig. 1:**
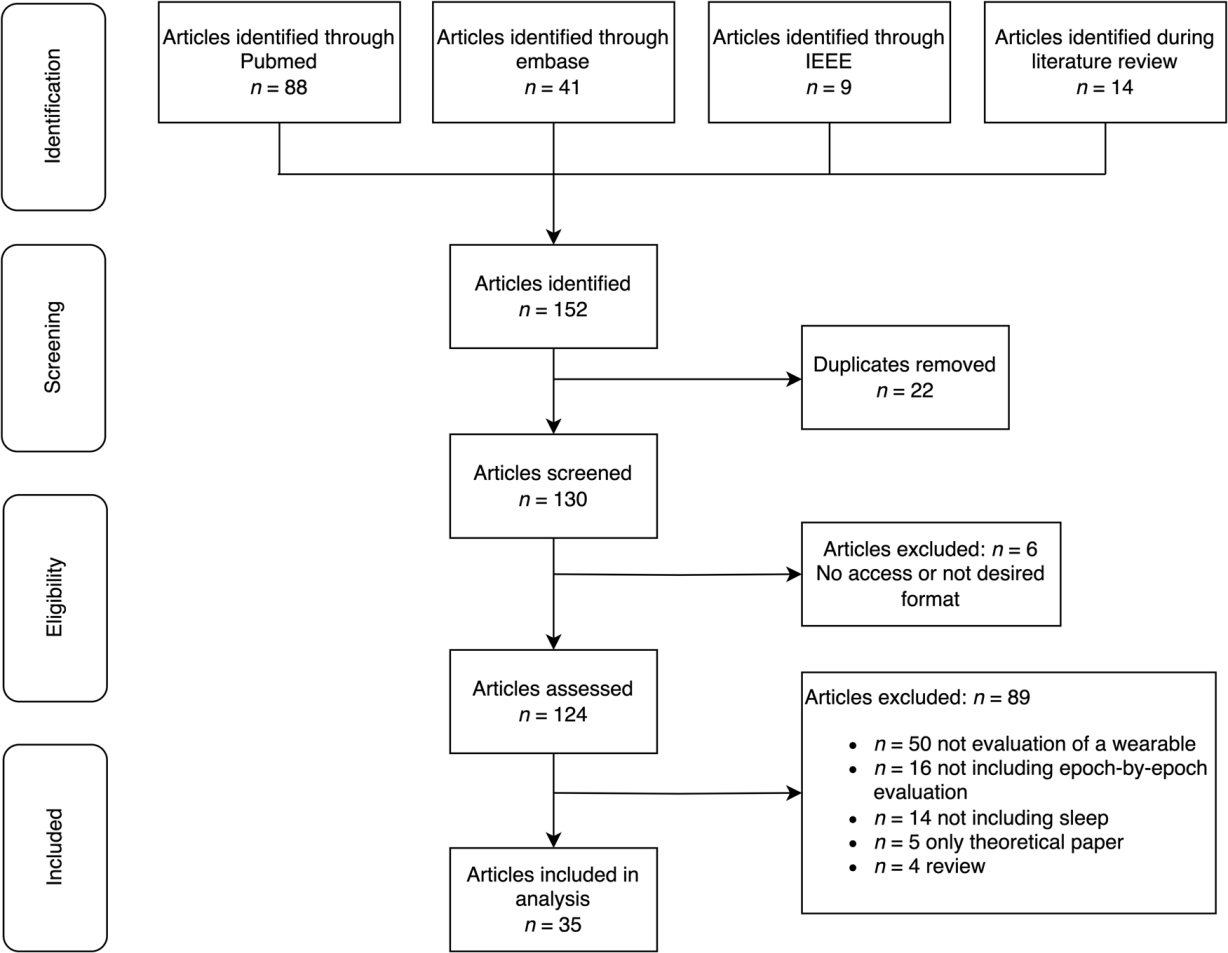
Search workflow depicting the identification, screening, eligibility, and inclusion of articles in the review. The figure illustrates the sequential steps involved in the systematic search process, including the identification of relevant articles, screening for eligibility criteria, and final inclusion of selected articles in the review.

This scoping review identified a total of 35 articles that evaluated a total of 62 setups of wearable devices, some of which occurred several times in different articles, as shown in Fig. 1. On PubMed 88 articles were identified, On Embase 41 articles were retrieved and on IEEE Xplore 9 articles. While screening through the articles, an additional 14 relevant articles were identified. While screening 22 duplicates and six inaccessible or incompatible articles were removed, leaving a total of 124 articles for evaluation. Fifty articles were excluded either did not discuss wearables or did not assess them, and another 14 articles did not evaluate the sleep metrics of the wearables. Additionally, 4 review articles and 5 theoretical articles were removed. Finally, 16 articles were removed where no epoch-by-epoch evaluation was included, resulting in 35 articles that were deemed suitable for in-depth analysis. Five of which were analyzed in more depth to extract the details for sleep staging algorithms and the used features. It was observed that the trend in wearable technology is shifting toward multi-sensor devices, where wearables incorporate not only accelerometers but also PPG, temperature, or other types of sensors. Specifically, this review included 62 wearable setups, of which 28 exclusively utilized accelerometers and 32 incorporated multiple different sensors. For two devices^16,17^ it was not clearly stated what sensor input(s) are being used to assess sleep.

### Characteristics of participants

Sleep stages exhibit significant variation both between males and females and across different age groups.^18^ Most of the studies included a relatively balanced number of male and female participants, except Fedorin et al.^19^ did not state the gender distribution. Eight studies focused on children and adolescents^17,20–26^, and five studies targeted young adults^27–31^, which was defined as articles reporting an average age below 25 or specifically stating that they investigated young adults. Only two articles^32,33^ examined the performance of wearable devices in an older population, meaning having an average age over 50. One article also had an average investigated age above 50 but reported a large variance in age^34^. The remaining 22 studies covered mainly individuals between 25 and 50 years. Finally, Fedorin et al.^19^ did not state the age of their participants.

### Inclusion of participants with sleep disorders and/or comorbidities

**Fig. 2:**
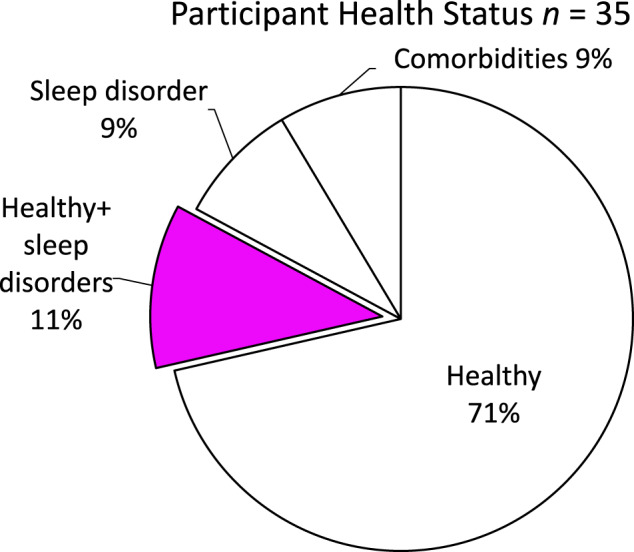
Distribution of included participants based on health status per article. The figure presents the distribution of participants included in the reviewed studies based on their health status. Notably, only 11% of all included studies assessed the performance of wearables for sleep staging in both healthy participants and participants with sleep disorders.

Medical conditions like insomnia, sleep disorders, or neurological disorders can also affect sleep staging.^35^ The majority (25) of the included articles recorded data from healthy participants only. Four articles included healthy participants as well as participants with some kind of sleep disorder^25,32,36,37^. Three studies focused exclusively on participants with sleep disorders^20,34,38^. One article included only participants with unipolar major depressive disorder^39^, while another one only involved participants with dermatitis^40^. Finally, one article included only participants who had obstructive sleep apnea (OSA) had neurological disorders, and/or used medications that are known to have effects on sleep^33^. In Fig. 2 these findings are summarized.

### Types of devices and reference systems

**Fig. 3:**
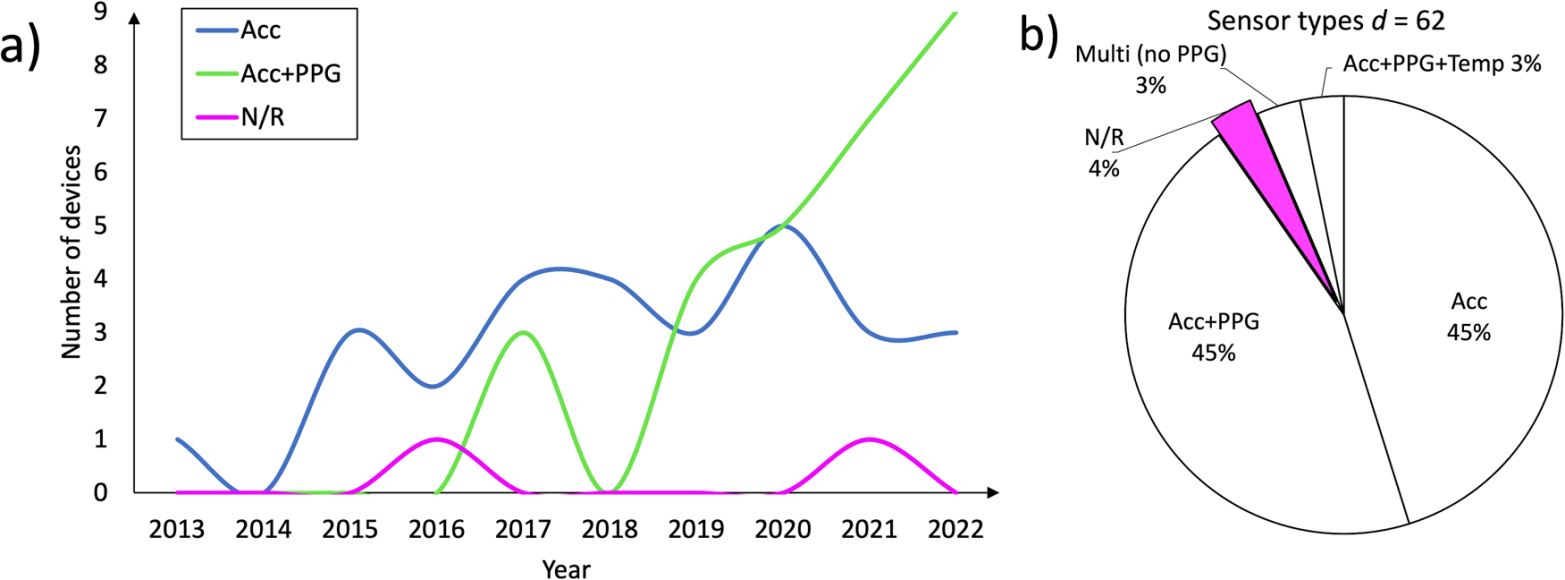
Type of sensors used to perform the sleep analysis per device. **a** A clear trend is visible that more wearable setups are investigated that include PPG data in sleep staging. **b** For 4% of all included device setups, it was not clear what sensor input the wearable used to do sleep staging. Acc Accelerometer data, PPG Photoplethysmography, Temp Temperature data, Multi (no PPG) Multi-sensor devices not including PPG, `*d*' refers to a wearable setup, while `N/R' stands for `not reported'.

The majority of devices examined in this review (*d* = 28, ‘*d*’ is the number of devices) relied solely on accelerometer data for sleep analysis. However, there has been an increasing trend in recent years towards utilizing both accelerometer and PPG data for evaluating sleep, which is reflected in the inclusion of 28 such devices in this review, as seen in Fig. 3. Further, two devices^41,42^ included in this review incorporated data from three sensors—accelerometer, PPG, and temperature sensors. An additional two devices^40,43^ utilized input from accelerometers and additionally other sensors, such as ambient light, bio-impedance, or skin temperature, but did not include a PPG sensor. Lastly, there were two devices^16,17^ for which the specific sensor input utilized for sleep analysis was not reported for all included devices.

On average, sleep/wake classification accuracies were reported to be 87.2% based on 53 assessed devices. There was no significant difference in accuracies between devices using only accelerometer data (86.7%, *d* = 28) and devices using both PPG and accelerometer data (87.8%, *d* = 22), as determined by a t-test (significance threshold *p* < 0.05). All reported accuracies ranged from 79% to 96%, except for Kanady et al.’s study^28^, which reported lower values of 54% and 64%. This difference can be attributed to their 24-hour measurement, which had a higher wake-to-sleep ratio compared to overnight measurements in other studies. Therefore, these accuracies reflect the generally poor performance of sleep classifiers in detecting wake. The average accuracy for 3-stage classification (wake vs. NREM vs. REM) was 69.7% (*d* = 3), and for 4-stage classification (wake vs. light vs. deep vs. REM), it was 65.2% (*d* = 9). More detailed information is in Table 1.

**Table 1:** Overview of all articles included in this review

	Subjects			Data acquisition					Algorithm			Epoch-by-Epoch evaluation		
Author (year)	Number of participants	Health status	Age	Device	Aquisition site	Used sensors (ACC / PPG / …)	Reference measurement	Environment	Type (Proprietary/self-written)	Categories for sleep classification	Epoch length	2 stages (wake/sleep)	3 stages (wake/light/deep OR wake/NREM/REM)	4 stages (wake/light/deep/REM)
^38^ Dong et al. (2022)	37 (20 female)	Insomnia disorder	49 ± 2 years	Fitbit Charge 4	Nondominant wrist	ACC, PPG	PSG	Laboratory	Proprietary algorithm (normal setting)	Wake, light, deep, REM	30s	Accuracy: 87%	N/R	N/R
				Actiwatch Spectrum Pro	Nondominant wrist	ACC			Proprietary algorithm (medium threshold)	Wake, sleep	30s	Accuracy: 87%	N/R	N/R
^47^ Miller et al. (2022)	53 (26 female)	Healthy	25 ± 6 years	Apple Watch S6	Wrist	ACC, PPG	PSG	Laboratory	Proprietary algorithm	Wake, light, deep	5min	Accuracy: 88%	Accuracy: 53%	N/R
				Garmin Forerunner 245	Wrist	ACC, PPG			Proprietary algorithm	Wake, light, deep, REM	60s	Accuracy: 89%	N/R	Accuracy: 50%
				Polar Vantage V	Wrist	ACC, PPG			Proprietary algorithm	Wake, light, deep, REM	30s	Accuracy: 87%	N/R	Accuracy: 51%
				Oura ring 2nd gen	Finger	ACC, PPG			Proprietary algorithm	Wake, light, deep, REM	5 min	Accuracy: 89%	N/R	Accuracy: 61%
				WHOOP strap, generation 3	Wrist	ACC, PPG			Proprietary algorithm	Wake, light, deep, REM	30s	Accuracy: 86%	N/R	Accuracy: 60%
^42^ Ghorbani et al. (2022)	58 (26 female)	Healthy	37 ± 13 years	Oura ring 2nd gen, additional memory	One on each hand on finger which has best fit	ACC, PPG, temperature	PSG	Home	Proprietary algorithm (3rd generation, not finalized version)	Wake, light, deep, REM	30s	Accuracy: 93%	N/R	Accuracy: 76%
^31^ Chinoy et al. (2022)	21 (12 female)	Healthy	29 ± 5 years	Fatique Science Readiband Version 5	Together with fitbit on other arm	ACC	Dreem 2 (mobile EEG), research version dreem: 30s epoch, all sleep stages	Home	Proprietary algorithm	Wake, sleep	60s	Accuracy: 90%	N/R	N/R
				Fitbit Inspire HR	Together with readiband on other arm	ACC, PPG			Proprietary algorithm	Wake, light, deep, REM	30s	Accuracy: 89%	N/R	N/R
				Oura ring 2nd gen	Non-dominant hand, ring finger	ACC, PPG			Proprietary algorithm	Wake, light, deep, REM	30s	Accuracy: 90%	N/R	N/R
				Polar Vantage V Titan	Together with actiwatch on one arm	ACC, PPG			Proprietary algorithm	Wake, light, deep, REM	60s	Accuracy: 92%	N/R	N/R
				Actiwatch 2	Together with polar watch on one arm	ACC			Proprietary algorithm (medium threshold, Cole-kripke or Sadeh)	Wake, sleep	30s	Accuracy: 90%	N/R	N/R
^16^ Chinoy et al. (2021)	34 (22 female)	Healthy	28 ± 4 years	Actiwatch 2	Nondominant wrist	ACC	PSG	Laboratory	Proprietary algorithm (medium threshold)	Wake, sleep	30s	Accuracy: 89%	N/R	N/R
				Fatigue Science Readiband	Wrist (only subset of below devices worn)	N/R			Proprietary algorithm	Wake, sleep	60s	Accuracy: 88%	N/R	N/R
				Fitbit alta HR	Wrist	ACC, PPG			Proprietary algorithm	Wake, light, deep, REM	30s	Accuracy: 90%	N/R	N/R
				Garmin Fenix 5S	Wrist	ACC, PPG			Proprietary algorithm	Wake, light, deep, REM	60s	Accuracy: 88%	N/R	N/R
				Garmin Vivosmart 3	Wrist	ACC, PPG			Proprietary algorithm	Wake, light, deep, REM	60s	Accuracy: 88%	N/R	N/R
^40^ Mahadevan et al. (2021)	33 (23 female)	Atopic dermatitis patients	31 ± 16 years	GeneActiv Original	On both wirsts	ACC, ambient light, skin temperature	PSG	Laboratory	Self developed sleep detection pipline	Wake, sleep	30s	left/right Accuracy: 85/85%	N/R	N/R
^25^ Menghini et al. (2021)	39 (22 female)	27 Healthy 12 Insomnia	18 ± 1 years	Fitbit charge 3	Dominant wrist	ACC, PPG	PSG	Laboratory	Proprietary algorithm	Wake, light, deep, REM	30s	N/R	N/R	N/R
^30^ Miller et al. (2021)	6 (3 female)	Healthy	23 ± 2 years	WHOOP strap, generation 2	Nondominant wrist	ACC, PPG	PSG	Laboratory	Proprietary algorithm	Wake, light, SWS, REM	30s	N/R	N/R	N/R
				Actical z-series	Nondominant wrist	ACC			Proprietary algorithm (medium threshold)	Wake, sleep	30s	N/R	N/R	N/R
^49^ Stucky et al. (2021)	62 (35 female)	Healthy	34 ± 8 years	Fitbit charge 2	Nondominant wrist	ACC, PPG	PSG AASM (20s epochs)	Home	Proprietary algorithm (sensitive setting)	Wake, light, REM, deep	30s	N/R	N/R	N/R
^26^ Chee et al. (2021)	53 (28 female)	Healthy	15–19 years	Oura ring 2nd gen	Finger with best fit	ACC, PPG	PSG	semi-laboratory	Proprietary algorithm	Wake, light, deep, REM	30s	Accuracy: 89%	N/R	N/R
				Actiwatch 2	Nondominant wrist	ACC			Proprietary algorithm (both thresholds)	Wake, sleep	30s	Accuracy: 90–91%	N/R	N/R
^41^ Altini and Kinnunen (2021)	Dataset 1 59 (30 female) Dataset 2 19 (11 female) Dataset 3 40 (24 female)	Healthy	Dataset 1 16 ± 1 years Dataset 2 39 ± 9 years Dataset 3 45 ± 15 years	Oura ring 2nd gen (research device)	Finger with best fit	ACC,PPG, temperature	PSG	Laboratory or home	Different classifiers in comparison, using a Light Gradient Boosting Machine (LightGBM) classifier	Wake, light, deep, REM	30s	Accuracy: 96%	N/R	Accuracy: 79%
^28^ Kanady et al. (2020)	18 (13 female)	Healthy	27 ± 3 years	Basis B1	Nondominant wrist	ACC, PPG	PSG	Laboratory	Proprietary algorithm	Wake, light, deep, REM	30s	Accuracy: 54%	N/R	N/R
				Micro motionlogger	Nondominant wrist	ACC			Cole-kripke algorithm	Wake, sleep	60s	Accuracy: 64%	N/R	N/R
^48^ Roberts et al. (2020)	8 (3 female)	Healthy	41 ± 5 years	Apple watch series 2	Nondominant wrist	ACC, PPG	PSG	Laboratory	Proprietary algorithm and self-written	No staging	N/R	N/R	N/R	N/R
				Oura Ring 1st gen	Best fitting finger	ACC, PPG			Proprietary algorithm and self-written	Wake, light, deep, REM	30s	Accuracy: 90%	N/R	N/R
				ActiGraph Link	Dominant wrist	ACC			Proprietary algorithm (medium threshold)	Wake, sleep	60s	Accuracy: 88%	N/R	N/R
				Philips respironics spectrum plus	Nondominant wrist	ACC			Proprietary algorithm	Wake, sleep	30s	Accuracy: 90%	N/R	N/R
^29^ Miller et al. (2020)	12 (6 female)	Healthy	23 ± 3 years	WHOOP 2.0	Nondominant wrist	ACC, PPG	PSG	Laboratory	Proprietary algorithm (Generation 3.0)	Wake, light, SWS, REM	30s	Accuracy: 89%	N/R	Accuracy: 64%
^24^ Godino et al. (2020)	26 (13 female)	Healthy	10 ± 1 years	Fitbit charge HR	Nondominant wrist	ACC, PPG	PSG	Home	Proprietary algorithm	Wake (restless + wake), sleep	60s	Accuracy: 92%	N/R	N/R
^43^ Devine et al. (2020)	8 (4 female)	Healthy	30 ± 3 years	Zulu watch	Nondominant wrist	ACC, on-wrist detection	PSG	Laboratory	Proprietary algorithm	Wake, restless, light, deep	2 min	Accuracy: 90%	N/R	N/R
				Actiwatch 2	Nondominant wrist	ACC			Proprietary threshold (medium threshold)	Wake, sleep	30s	Accuracy: 91%	N/R	N/R
^33^ Regalia et al. (2020)	46 (21 female)	OSA, neurological, medications (bezodiazepine, beta blocker, SSRI, Donepezil)	66 ± 10 years	E4 wristband, Empatica	Nondominant wrist	ACC	PSG	Home	Self written actigraphy algorithm trained on previous data	Wake, sleep	30s	Accuracy: 81%	N/R	N/R
									Sadeh’s algorithm	Wake, sleep	30s	Accuracy: 79%	N/R	N/R
^23^ Lee et al. (2019)	58 (28 female)	Healthy	17 ± 1 years	Fitbit alta HR	Nondominant wrist	ACC, PPG	PSG	Quasi laboratory	Proprietary algorithm	Wake, light, deep, REM	30s	Accuracy: 90%	N/R	N/R
				Philips respironics actiwatch 2	Nondominant wrist	ACC			Proprietary algorithm (medium/high threshold)	Wake, sleep	30s	Accuracy: 93–94%	N/R	N/R
^51^ Walch et al. (2019)	31 (21 female)	Healthy	29 ± 9 years	Apple watch series 2 and 3	Wrist	ACC, PPG	PSG	Laboratory	Self-written classifier (Best performing classifier: Neural net)	Wake, NREM, REM	30s	Accuracy: 91%	Accuracy: 72%	N/R
^44^ Haghayegh et al. (2019a)	40 (17 female)	Healthy	27 ± 12 years	Motionlogger Micro Watch actigraphy	Nondominant wrist	ACC	single channel EEG (Zmachine Insight+) proprietary staging	Home	Different classifiers for actigraphy Best performing classifier: rescore Cole-Kripke	Wake, sleep	30s	Accuracy: 86%	N/R	N/R
^19^ Fedorin et al. (2019)	50	Healthy	N/R	Band-type wearable device (Samsung)	Wrist	ACC, PPG	PSG	Laboratory	Self-developed ML pipline	Wake,NREM, REM OR Wake, light, deep, REM	30s	N/R	Accuracy: 85%	Accuracy: 77%
^45^ Haghayegh et al. (2019b)	35 (17 female)	Healthy	27 ± 13 years	Fitbit Charge 2	2x, one on each wrist	ACC, PPG	Zmachine Insight+ 30s epochs (wake, REM, N1, N2, SWS) proprietary IA	Home	Proprietary algorithm	Wake, light, deep, REM	30s	Accuracy: 85%	N/R	N/R
				Motionlogger Micro Watch actigraphy	Wrist	ACC			Sadeh algorithm	Wake, sleep	30s	Accuracy: 85%	N/R	N/R
^52^ Pigeon et al. (2018)	20 (7 female)	Healthy	30 ± 13 years	MyCadian	Nondominant wrist	ACC	PSG	Laboratory	Proprietary algorithm	Wake, sleep	30s	Accuracy: 91%	N/R	N/R
				Actiwatch 2	Nondominant wrist	ACC	PSG		Proprietary algorithm (medium threshold)	Wake, sleep	30s	Accuracy: 88%	N/R	N/R
^22^ Pesonen and Kuula (2018)	Children 17 (9 female) Adolescents 17 (8 female)	Healthy	Children 11 ± 1 years Adolescents 18 ± 2 years	Polar fitness tracker (prototype of A370)	Nondominant wrist	ACC	PSG	Home	Proprietary algorithm	Wake, sleep	30s	Children Accuracy: 91% Adolescents Accuracy: 90%	N/R	N/R
				Actiwatch 2	Nondominant wrist	ACC			Proprietary algorithm (medium threshold)	Wake, sleep	30s	Children Accuracy: 90% Adolescents Accuracy: 89%	N/R	N/R
^39^ Cook et al. (2017)	21 (17 female)	Unipolar major depressive disorder	27 ± 5 years	Fitbit Flex	Nondominant (left) wrist	ACC	PSG	Laboratory	Proprietary algorithm (normal setting)	Wake, sleep	60s	Accuracy: 88%	N/R	N/R
				Actiwatch 2	Nondominant (left) wrist	ACC			Proprietary algorithm (medium threshold)	Wake, sleep	30s	Accuracy: 87%	N/R	N/R
^37^ Kuo et al. (2017)	81 (34 females)	56 good, 25 poor sleep quality	28 ± 6 years	Accelerometer, selfmade, 1 Hz sampling rate, 10 bit resolution, ± 3g	Left wrist	ACC	PSG (Rechtschaffen and Kales rules)	Laboratory	Self developed algorithm for sleep staging	Wake, sleep	30s	Accuracy: 92%	N/R	N/R
^34^ Razjouyan et al. (2017)	21 (10 female)	Self-reported sleep problems	50 ± 13 years	Actiwatch-L CamNtech Ltd	Wrist, dominant hand	ACC	PSG	Laboratory	Proprietary algorithm	Wake, sleep	60s	Accuracy: 80%	N/R	N/R
^50^ Beattie et al. (2017)	60 (24 female)	Healthy	34 ± 10 years	2x Fitbit surge	On each hand one	ACC, PPG	PSG	Home or Hotel	Self-written classification	Wake, light, deep, REM	30s	N/R	N/R	Accuracy: 69%
^21^ de Zambotti et al. (2017a)	41 (13 female)	Healthy	17 ± 2 years	Oura ring 1st gen	Finger nondominant hand with best fit	ACC, PPG	PSG	Laboratory	Proprietary algorithm (first version)	Wake, light, deep, REM	30s	N/R	N/R	N/R
^53^ de Zambotti et al. (2017b)	44 (26 female)	Healthy	35 ± 12 years	Fitbit charge 2	Nondominant wrist	ACC, PPG	PSG	Laboratory	Proprietary algorithm	Wake, light, deep, REM	30s	N/R	N/R	N/R
^20^ Toon et al. (2016)	78 (27 female)	Suspected obstructive sleep apnea	8 ± 4 years	Jawbone UP (first release)	Nondominant wrist	ACC	PSG	Laboratory	Proprietary algorithm	Wake, sleep	60s	Accuracy: 86%	N/R	N/R
				Actiwatch 2	Nondominant wrist	ACC			Proprietary algorithm (medium wake threshold)	Wake, sleep	60s	Accuracy: 87%	N/R	N/R
^17^ de Zambotti et al. (2016)	32 (15 female)	Healthy	17 ± 3 years	Fitbit Charge HR	Nondominant wrist	N/R	PSG	Laboratory	Proprietary algorithm	Wake (=wake and restless), sleep	60s	Accuracy: 91%	N/R	N/R
^32^ de Zambotti et al. (2015)	28 (28 female)	12 insomnia disorder	50 ± 4 years	Jawbone UP	Wrist	ACC	PSG	Laboratory	Proprietary algorithm	Wake, sleep	60s	N/R	N/R	N/R
^27^ Slater et al. (2015)	108 (51 female)	Healthy	23 years	GTX3+	Nondominant wrist	ACC	PSG	Laboratory	Proprietary algorithm (Sadeh’s algorithm)	Wake, sleep	60s	Accuracy: 84%	N/R	N/R
				GTX3+	Hip	ACC			Proprietary algorithm (Sadeh’s algorithm)	Wake, sleep	60s	Accuracy: 86%	N/R	N/R
^36^ Marino et al. (2013)	77 (30 female)	Healthy or chronic insomniac	35 ± 13 years	AW-64, Minimitter or actiwatch spectrum	Wrist	ACC	PSG (Rechtschaffen and Kales rules)	Laboratory	Proprietary algorithms	Wake, sleep	30s	Accuracy: 86%	N/R	N/R

*ACC* accelerometer sensor, *PPG* Photoplethysmography sensor, *PLMD* Periodic Limb Movement Disorder, *NREM* non-REM sleep stages. PSG data is evaluated according to the AASM manual in 30s epochs unless otherwise stated.

**Fig. 4:**
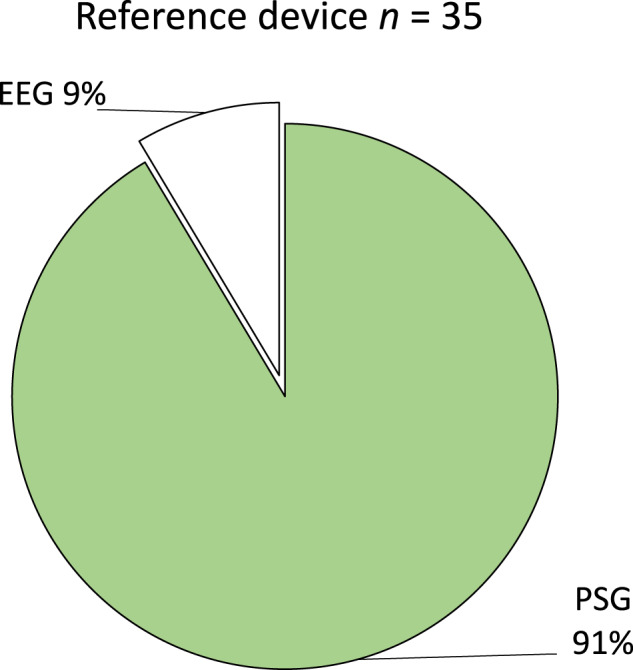
Ground truth methods used for evaluating wearables per article. PSG is the most used reference device, used in 91% of all identified articles. Note `*n*' refers to number of articles.

Articles discussed data collection at sleep laboratories (*n* = 23, ‘*n*’ is the number of articles), at home (*n* = 9) or quasi-/semi-laboratories (*n* = 2). One study included recordings from particpants’ home and a sleep laboratory^41^. Most of the articles (*n* = 32) used PSG as a reference system to validate the results of the wearables, as it can be seen in Fig. 4. However, three studies utilized an EEG system^31,44,45^ as a reference, two used a single-channel EEG device^44,45^ and one used the Dreem 2^31^ mobile EEG device.

### Sleep staging epoch lengths

**Fig. 5:**
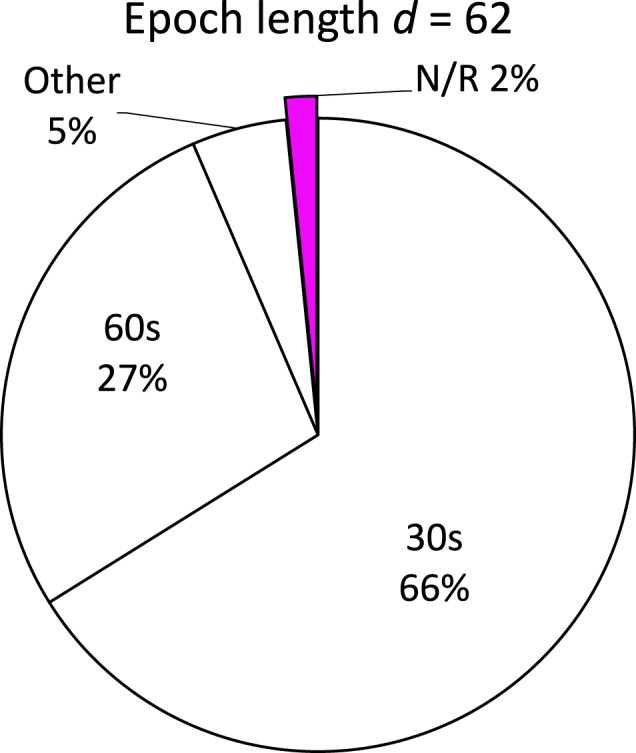
Reported length of epochs used to evaluate the performance of wearables per wearable setup. In 66% of the included wearable setups, the standard epoch length of 30 seconds was used. Other: 2 min and 5 min epochs, `*d*' refers to a wearable setup, and `N/R' refers to `Not reported'.

According to the guidelines for sleep staging, the PSG data are analysed in 30-s segments, called epochs, and these are then classified into the sleep stages^46^. About two thirds (*d* = 41) of the 62 wearable setups in the reviewed articles provided epochs of 30 s, which can be directly compared to the epochs of the PSG data. A quarter (*d* = 17) of the wearable setups had access to 60-s epochs. One article^43^ employed a device that only provided access to 2-min segmented data. Furthermore, for two devices in one study^47^ the sleep stages in epochs of 5 min were reported. For one device^48^ the epoch length was not stated. The distribution of epoch lengths used can be seen in Fig. 5.

A challenge is to compare sleep stages that are half or two/four/ten times as long as the reference measurements. A commonly used method for 60-s epochs is to fuse the PSG epochs to 60 s. If one or both epochs are classified as wake, they are scored as wake, and if both are classified as sleep, they are scored as sleep^17,20,24,27,28,32,34^. Another commonly used method is to split the epochs into 30-s segments and assign them the same value as the long epoch^31,39,47^. Roberts et al.^48^ used the timestamp of the beginning of the staged epoch and used the classification of the reference epoch with the nearest start timestamp; no conversion between 30 s and 60 s occurred. Devine et al.^43^ assigned sleep and wake with the values 1 and 0, respectively, averaged the values over four epochs, and then rounded to the nearest integer to obtain 2-min epochs. Chinoy et al.^16^ scored the PSG data at 30-s and 60-s epochs to be able to compare it to devices with 30-s epochs and devices with 60-s epochs. Stucky et al.^49^ used PSG data that was scored in 20-s epochs and compared it to 30-s epochs where they looked at the PSG intervals and compared it to the dominating device stage in that interval; if two were equal, the first one was chosen.

When authors were able to work with 30-s epochs (or raw data) of commercially available devices, the devices often had to be provided by the company or the authors were employed by the company^21,22,26,29,30,33,41,42,47^.

### Algorithms for sleep staging

**Fig. 6:**
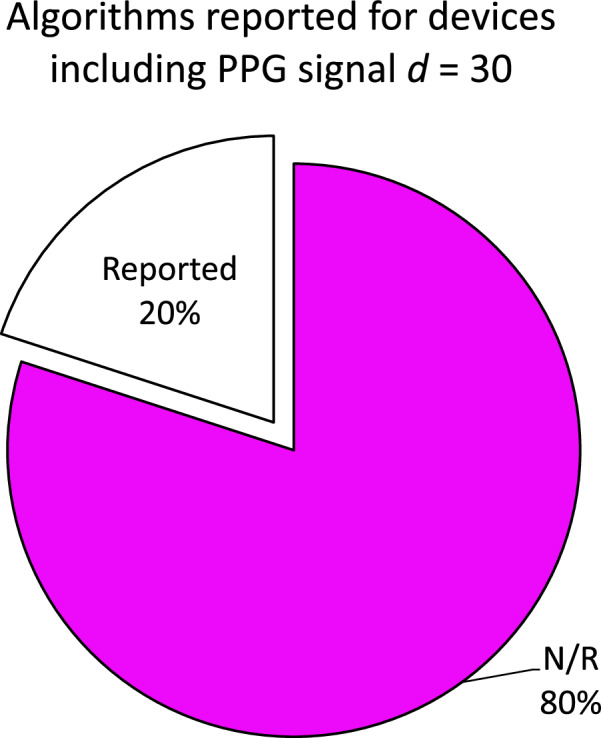
Reported algorithms for devices using PPG sensors. The figure depicts the percentage of devices utilizing PPG sensors and the corresponding reported algorithms used for sleep staging. Notably, only 17% of all devices including PPG signals reported the algorithm used for sleep staging. Note `*d*' refers to a wearable setup, while `N/R' stands for `not reported'.

The majority of the articles^16,17,19,20,22,24–32,36–39,42,43,45,47–53^ included in this review reported their findings based on proprietary algorithms used by wearable device companies, with many not disclosing the specific features employed in their sleep staging algorithms, as it can be seen from Fig. 6. For sleep detection using only accelerometer data (actigraphy), well-established algorithms are most often used, including the Cole-Kripke algorithm^54^, the University of California, San Diego (UCSD) scoring algorithm^55^ and the Sadeh algorithm^56^. In general, they calculate weighted sums of activity levels in one-minute intervals, including levels from preceding and succeeding minutes^57^. For devices using also PPG data, five articles^19,41,48,50,51^ describe their own sleep staging algorithms in detail using machine learning, which are reviewed in the following sections. Further Mahadevan et al.^40^ described a possible algorithm for a wake / sleep detection using accelerometer data, skin temperature and an environment light sensor but no PPG data.

The evaluated classifiers for sleep staging with wearable devices include linear discriminant classifier, quadratic discriminant classifier, random forest classifier, support vector machine, neural nets, logistic regression, k-nearest neighbor and gradient boosting machine^19,41,48,50,51^. The overall best accuracy for sleep/wake classification has been shown to be 96% with the light gradient boosting machine^41^. The best accuracy for 3 stages sleep staging was 85%^19^ with the linear discriminant classifier. The overall highest accuracy for 4 stage sleep staging was 79%^41^ with the light gradient boosting machine. It has to be mentioned, that both Beattie et al.^50^ and Walch et al.^51^ state in their articles that the choice of the classifier was not as impactful as the selection of the input features.

### Data processing and feature selection

In some studies^19,41,50^ before feature extraction for classifier training, the data underwent pre-processing. This included peak detection in PPG to estimate RR intervals in ECG^50^ or detrending, denoising, and filtering on all raw data^19^. Altini and Kinnunen^41^ applied a 5th order Butterworth filter (3–11 Hz) on the accelerometer data and performed temperature artifact rejection by masking values outside of 31–40 degrees. They applied a real-time moving average filter to the PPG data and removed intervals more than 16 bpm away from the 7-point median of its immediate neighbors. Additionally, they required the existence of five consecutive windows.

Beattie et al.^50^ used accelerometer features including an integration of the accelerometer signal in 30-s epochs, the magnitude (maximum and minimum of each axis), and time since the last movement and until the next significant movement. Walch et al.^51^ described their feature extracted from the accelerometer as the activity count from the raw data, which should be similar to the features used by actigraphy (described and evaluated by te Lindert et al.^58^). Altini and Kinnunen^41^ included the trimmed mean, maximum, and interquartile range of each axis in 30-s windows. Furthermore, the mean amplitude deviation and the difference in arm angle were evaluated of 5-s epochs and then aggregated to 30-s epochs. Finally, Fedorin et al.^19^ also utilized features derived from accelerometer data, but their specific features were not explicitly stated.

The included features derived from the PPG measurements varied greatly from article to article. Beattie et al.^50^ extracted heart rate (HR) from the PPG signal and used several heart rate variability (HRV) features in their sleep staging classifier, including high frequency (HF), low frequency (LF), and very low frequency (VLF) power, root mean sum of squared distance (RMSSD), percentage of adjacent RR intervals differing by more than 50 ms (pNN50), delta RR, mean HR, 90th percentile HR, and 10th percentile HR. They also included breathing rate features such as HF power (0.15–0.4 Hz), LF power (0.04–0.15 Hz), and VLF power (0.015–0.04 Hz). Altini and Kinnunen^41^ used several HRV features in their sleep staging classifier, including HR, RMSSD, standard deviation of normal-to-normal intervals (SDNN), pNN50, LF power (0.04–0.15 Hz), and HF power (0.15–0.4 Hz), frequency peak in LF and HF, total power, normalized power, breathing rate, mean, and coefficient of variation of zero-crossing interval. On the other hand, Walch et al.^51^ used the bpm values for every second and the standard deviation of the windows around the scored epoch. Finally, Fedorin et al.^19^ included the HRV and the RR in its time and frequency domains and in nonlinear time sequence processing. They also used some PPG shape features, although these were not specified.

In their classifier, Walch et al.^51^ incorporated a feature termed “clock proxy,” which is a cosine wave derived from an individual’s circadian clock that was estimated using data from the previous night’s sleep with the wearable. Fedorin et al.^19^ included statistical information regarding sleep stages as features, such as a sleep stage transition probability matrix and the probability of each sleep stage occurring per hour after falling asleep. Altini and Kinnunen^41^ included features derived from a negative temperature coefficient sensor, including mean, minimum, maximum, and standard deviation, as well as a sensor-independent circadian factor. The circadian factor is composed of a cosine wave representing circadian drive, a decay representing the decay of homeostatic sleep pressure, and a linear function representing the elapsed time since the beginning of sleep.

Altini and Kinnunen^41^ did a normalization of most of the features per night, excluding some acceleration features, and then used them as an input for the models. Beattie et al.^50^ used a set of rules after sleep staging to penalize unlikely physiological patterns.

### Sleep staging without full raw data access

In the study by Roberts et al.^48^, already processed data provided by Apple and Oura were used to distinguish between wake and sleep without full raw data access, like the previously described classifiers. The Apple Watch Series 2 provided raw accelerometer data but only provided access to bpm estimates for the heart rate, sampled at approximately 0.2 Hz. For the Oura Ring, the researchers used motion counts provided every 30 s and RR intervals from the PPG sensor. They employed a gradient boosting classifier and achieved accuracy and sensitivity comparable to the proprietary sleep staging algorithm used by Oura. At the time of this study, Apple did not yet have its own sleep classifier. The model trained on the data obtained from these devices achieved higher accuracy for the Apple Watch than for the Oura Ring. The researchers suspected that the difference in accuracy and specificity could be attributed to the various types of data available from the devices. Additionally, the algorithm developed in this study was suitable for real-time applications.

### Influence of different features on classifier performance

The reported specificities for sleep/wake detection range from 41% to 60.2% (accuracies 90%/92.6%)^48^ for the algorithms using already processed data and 65% (sensitivity fixed at 90%)^51^ up to 80.74% (accuracy 98.15%)^41^. Walch et al.^51^ stated that for the wake/sleep staging, the motion features are a good predictor, and the addition of the circadian features increases the accuracy more than the addition of the heart rate features. Altini and Kinnunen^41^ also used motion as the baseline accuracy and added features, reporting that the addition of temperature and HRV increased the accuracy by about the same amount, while the last added circadian features only increased the f1 score. Roberts et al.^48^ found that the specificity could be increased by around 20–35% when the wake epochs are oversampled, at the cost of 8–12% of accuracy.

The reported accuracies for three-stage sleep staging were 69%^51^ and 85%^19^, with Cohen’s kappa values ranging from 0.4 to 0.67 indicating moderate to substantial agreement with the PSG sleep staging. Walch et al.^51^ found that motion is the weakest predictor of three-stage sleep staging, indicating that heart rate features are much more important.

For four-stage sleep staging, the reported accuracies were 69%^50^, 77%^19^ and 79%^41^ and the Cohen’s kappa values were 0.52^50^ and 0.58^19^, indicating moderate agreement with the PSG sleep staging. Beattie et al.^50^ stated that the Cohen’s kappa value is the same if one is only using motion or accelerometer features and that the score doubles when using both feature types. Altini and Kinnunen^41^ started with a baseline accuracy using just motion features, resulting in an accuracy of 57%. The addition of temperature features added 4%, while the addition of HRV features increased accuracy by 16%. Finally, the addition of circadian features resulted in an increase in accuracy by 3%.

## Discussion

The objective of this review was to assess the current literature on the challenges associated with algorithm development in sleep staging using wearables. To achieve this, we conducted an extensive search to identify previous research in this area. Although many articles discussed wearables and sleep evaluation, most focused on sensing technologies or devices that only use accelerometer data. Despite the growing number of wearables that incorporate multiple sensors for sleep staging, there is a lack of research on algorithms used for sleep staging and the potential benefits of using multi-sensor inputs.

The American Academy of Sleep Medicine (AASM) expressed the need for validation of consumer sleep technologies^59^. However, there are no standardized protocol or measures for evaluating wearable devices which do not include EEG sensors. Menghini et al.^60^ proposed a framework to improve validation. Two types of assessment measures that are commonly used are: total duration of different sleep quality measures (total sleep time, sleep onset latency, wake after sleep onset, and sleep efficiency) and epoch-by-epoch sleep staging comparison (accuracy, sensitivity, and specificity). In this review only articles were included which report results of an epoch-by-epoch sleep staging comparison.

PSG is considered the gold-standard method for diagnosing sleep disorders. Physiological signals, including EEG, electrooculography (EOG), electromyography (EMG), and electrocardiography (ECG), are measured during PSG to identify sleep stages. Sleep is classified into N1, N2, N3, and REM stages, each with unique physiological patterns, according to the AASM sleep scoring^46^. The N1 and N2 stages are often combined and referred to as light sleep, whereas N3 is considered deep sleep. However, manual sleep staging may not be perfectly consistent across different scorers. The agreement among scorers for sleep staging ranged from 78.9%^61^ to 82.6%^62^. Before 2007 the standard to classify sleep stages was developed by Rechtschaffen and Kales^63^. In this standard the sleep is classified in S1 to S4, REM and movement time. Generally, S1 to S4 are referred to N1, N2 and N3 where S3+S4 refer to N3, and REM stays REM. Although significant differences between the two manuals have been identified^64^ and the usage of data of two different manual have to be handled carefully.

Sleep evaluation faces several limitations: PSG, the gold standard measurement device, is bulky and inconvenient, and existing studies using actigraphy, a widely used alternative, have shown limitations in detecting wake episodes and providing more detailed sleep staging. However, Ryser et al.^65^ have recently demonstrated a more reliable approach for correctly classifying wake epochs. New generations of wearables, with multiple sensors for PPG or temperature, aspire to overcome these limitations and provide more detailed sleep staging from unobtrusive devices using more advanced algorithms.

The current review acknowledges certain limitations that should be taken into consideration. Firstly, although a thorough search was conducted across three platforms (IEEE Xplore, PubMed, and Embase), it is important to note that there is a possibility of missing out on relevant articles. Secondly, some of the selected articles did not report accuracy as a primary outcome, but other results like sensitivity, specificity or total durations of sleep and wake. This may impact the overall representation of the findings in the final table, potentially influencing the interpretation of the results. These limitations, though present, do not undermine the value of this review, but rather highlight the importance of future research to report all outcome values and address any potential gaps to enhance our understanding of the topic.

We identified two main evaluation metrics for sleep wearables: total duration of sleep and wake time and epoch-by-epoch sleep classifier evaluation. These metrics are often reported in relation to PSG or EEG measurements and sometimes in combination with actigraphy devices. However, the reported metrics need to be treated with caution due to various sources of error, such as data synchronization issues and variable sleep staging epoch lengths. We decided to focus on articles reporting epoch-by-epoch results as these results contain the most information about the performance of classifiers.

Our in-depth analysis of the algorithms for sleep staging with multiple sensor inputs, especially the addition of PPG features to machine learning models, shows promising results. Feature selection has been shown to be crucial for the development of a sleep staging classifier. Next to features extracted from the accelerometer and the PPG data, some further features, such as temperature, were used. Additionally, features that were not from sensors, such as circadian features and statistical information, were included. A recent study^66^ demonstrated that the breathing rate can be extracted from an accelerometer positioned on the chest. This extracted breathing rate could be used as another feature for classifiers sleep staging classification.

However, most of the reviewed articles did not provide insight into the algorithms used for sleep staging, as they were proprietary algorithms provided by the manufacturer. This makes it hard to compare the same device in two different studies and may be a cause for differences. Furthermore, access to sleep staging epochs is often limited, and the authors of the articles had to rely on the manufacturer to provide them. Consequently, for many of the in-depth analysis articles, the data were provided by or associated with the manufacturer of the device.

While our primary focus is on wearables, it is essential to recognize that the field of sleep evaluation continues to evolve. Recent research has also evolved beyond traditional wearables, exploring sleep staging from sound analysis^67,68^. Although not within the scope of this article, sound-based sleep staging methods, which analyze audio data during sleep, offer a promising avenue for non-intrusive assessment of sleep quality and staging. Future studies might explore combinations between sound-based sleep monitoring and wearable technologies to further enhance the accuracy and comprehensiveness of sleep evaluation.

Further research and standardization of the framework^60^ are necessary to evaluate the benefits of including multiple sensors in wearables for reliable sleep staging. This requires access to epoch-by-epoch data and knowledge of the algorithms used. Moreover, a deeper understanding of the important features measured by wearables should be addressed. The data sets used should put special emphasis on heterogeneous field participants, including varying ages, different ethnicities, and a balanced gender distribution. Further emphasis should be placed on investigating the performance of wearables for sleep disorders and other comorbidities.

After conducting this literature review the following is recommended for future work:

- Conduct validation studies to evaluate algorithm performance, particularly when involving diverse participants with sleep disorders (like insomnia or sleep apnea) and comorbidities (like pychiatric disorders). Implementing equity, diversity and inclusion will enhance the generalizability of the findings and allows for a comprehensive assessment of the algorithm’s effectiveness in real-world scenarios. As it can be seen from Fig. 2, most of the studies were conducted with only healthy participants. The sample size of the articles reported in this review range from 6 to 118 participants. Where the average number of participant is 42.6. In order to achieve generalization it is important to have a reasonable large dataset which should contain more than 50 participants. In general we recommend using the article of Bujang and Adnan^69^ to calculate the suitable sample size.
- Compare commercially available multi-stage devices across studies to validate their performance. The validation process plays a pivotal role in ensuring the reliability and accuracy of multistage devices in detecting sleep stages, while also providing valuable insights into the performance of diverse algorithms. Through systematic evaluation across multiple studies, researchers can acquire a comprehensive understanding of the strengths, limitations, and areas for improvement of these devices. As it can be seen from the Table 1, only a fraction of all available wearables doing sleep staging have been validated in independent studies to validate their performance.
- Conduct investigations to thoroughly explore and understand the significant features measured by wearable sensors, such as accelerometer, PPG, temperature, and other non-sensor-based features. By delving into these features, researchers can gain insights into their respective contributions and potential synergies in assessing sleep quality and stages. Understanding the characteristics, strengths, and limitations of each sensor-based and non-sensor-based feature enables researchers to make informed decisions regarding their inclusion in algorithms and data analysis pipelines. The necessity for more investigation in features arise from the fact that only 20% of all articles reported the used algorithm (Fig. 6) and in total only 5 articles described the used features.
- Consistently report sensor specifications (type, resolution, measurement range), validation details (sensor input, epoch length) and performance metrics (accuracy, sensitivity, specificity) for transparency and comparisons^60^. For example, sleep data is typically more abundant than wake data in sleep studies, as individuals spend a significant portion of their time asleep. This data asymmetry could impose bias in the algorithm toward having a higher likelihood of correctly identifying sleep stages but may have more difficulty accurately classifying wakefulness. In the following unbiased metrics should be used to report the performance of a classifier, especially the Matthews correlation coefficient^70^.
- Cultivate the open-source availability of classifier code for independent validation and research collaboration. This facilitates rigorous peer review and enables researchers to in-depth check the algorithm’s methodology. It also allows other researchers to reproduce the results, conduct comparative analyses, and build upon existing work.

In conclusion, accurate and reliable consumer sleep technology is pivotal in comprehending sleep patterns and their impact on health. Our literature review uncovered an increasing trend in utilizing accelerometer and photoplethysmography (PPG) data for sleep assessment, with the integration of PPG features and additional sensors demonstrating enhanced sleep stage classification. To achieve precise sleep stage classification, meticulous analysis and optimization of data processing, alignment, epoch length, and feature selection are imperative. Collaborative endeavors between sleep researchers and device manufacturers are instrumental in refining machine learning models and augmenting the accuracy of sleep wearables. Further research is required to validate the performance of multi-sensor devices, deepen the understanding of key wearable-based features, and assess their efficacy in sleep disorders and comorbidities. Five recommendations for future work are proposed: (1) validate algorithms after implementing equity, diversity, and inclusion, (2) compare multi-stage device performance, (3) explore impact of features, (4) report validation use performance metrics consistently, and (5) promote open-source classifier and data availability. These guidelines could facilitate more precise and reliable sleep assessment, ultimately benefiting individuals’ well-being and advancing the field of sleep research.

## Methods

### Literature Search and Selection Criteria

We conducted a literature search across IEEE Xplore, PubMed, and Embase, adhering to PRISMA guidelines for systematic reviews^71^. The search covered publications from January 2013 to January 2023, focusing on recent developments in sleep assessment using wearable technology. Search terms included ‘sleep’, ‘quality’, ‘efficiency’, ‘assessment’, ‘evaluation’, ‘actigraphy’, ‘accelerometer’, ‘PPG’, ‘photoplethysmogram’, ‘photoplethysmography’, ‘heart rate’, and ‘wearable’. These terms were combined using Boolean operators to capture a broad range of relevant studies. The detailed search terms can be found in the supplemental material (see “Supplementary methods”). The literature review process involved one author (V.B.) conducting the initial search and a second author (M.E.) independently verifying the results.

Inclusion criteria for the review were articles presenting results of wearable devices for sleep evaluation on an epoch-by-epoch basis. Exclusion criteria included duplicate publications, inaccessible articles (lacking full-text availability), studies not relevant to wearable technology, those not assessing sleep metrics or lacking epoch-by-epoch evaluation, as well as review articles and theoretical papers.

### Data Analysis and Statistical Approach

For data analysis, we focused on the accuracy of sleep staging classifiers as reported in the selected studies. Given the potential imbalance in sleep stage datasets (disproportionate representation of sleep versus wake epochs), we chose accuracy for its widespread recognition and interpretability in sleep research. The analysis involved compiling reported accuracies of various devices and algorithms, specifically noting their performance in differentiating between sleep stages such as wake, NREM, REM, light sleep, and deep sleep.

A t-test was employed to assess statistically significant differences in classifier accuracies among the reviewed devices and algorithms. This involved calculating mean accuracy values for each device or algorithm and comparing them using the t-test, with a set significance level of *p* < 0.05. This statistical analysis aimed to identify any significant trends or disparities in the performance of various sleep staging technologies.

### Supplementary information

Supplemental Material

## Data Availability

The authors declare that all data supporting the findings of this study are available within this paper.
